# Pontine Osmotic Demyelination Syndrome in a Pregnant Woman With Sepsis and Wernicke’s Encephalopathy

**DOI:** 10.7759/cureus.73618

**Published:** 2024-11-13

**Authors:** Alizah Khan, Manal Khan, Shahab T Sharafat, Omar A Ali, Litty Paulose, Ayesha Aslam Dalvi

**Affiliations:** 1 Clinical Sciences Department, Dubai Medical College for Girls, Dubai, ARE; 2 Internal Medicine, Dubai Medical College for Girls, Dubai, ARE; 3 Neurology, University of Sharjah, Sharjah, ARE; 4 Internal Medicine, University of Sharjah, Sharjah , ARE; 5 Obstetrics and Gynecology Department, Latifa Women and Children Hospital, Dubai, ARE

**Keywords:** maternal malnutrition, non-alcoholic wernicke's encephalopathy, osmotic demyelination syndrome (ods), pontine osmotic demyelination syndrome, septic miscarriage

## Abstract

A pregnant woman was brought to the emergency department looking starved and neglected. She was diagnosed with sepsis and started on intravenous antibiotics. She was also disoriented and hypernatremic. When the fetal heart sounds were found to be absent, the patient was diagnosed with septic miscarriage, which was managed by misoprostol, and after the expulsion of the products of conception, she was taken for evacuation of the uterus under anesthesia. Her brain CT was normal; however, due to her disorientation and altered mental status, a brain MRI was done, which revealed findings suggestive of Wernicke's encephalopathy and pontine osmotic demyelination syndrome (ODS), for which she received intravenous fluids, high doses of vitamins B, C and D, as well as other supplements like calcium, magnesium, and zinc. She received nasogastric feeding as she had difficulty swallowing and physiotherapy for the weakness in her upper and lower limbs. The patient was stable on discharge with good oral intake and was able to mobilize with a wheelchair.

## Introduction

Osmotic demyelination syndrome (ODS) was initially delineated in 1959 by Lambeck et al. following their observation of four patients with various degrees of quadriplegia, encephalopathy, and pseudobulbar palsy who were found to have pontine myelinolysis. Initially, these manifestations were attributed to alcoholism and malnutrition [1]. However, in 1976, Tomlinson et al. elucidated that similar symptomatology could arise independent of malnutrition or alcoholism, implicating rapid correction of hyponatremia as a causative factor [2]. Further research has consistently highlighted the rapid correction of severe hyponatremia as the primary mechanism causing central pontine myelinolysis (CPM) [3]. Over time, an increasing number of cases associated with central pontine myelinolysis and extrapontine myelinolysis (EPM) were documented in scientific literature. Spotting their shared pathophysiological mechanisms, the scientific community decided to amalgamate these conditions under the label of "osmotic demyelination syndrome" [1].

Patients with ODS typically present due to rapidly corrected hyponatremia; however, scarce reports have been made concerning ODS resulting from the rapid correction of hypernatremia [4]. The pathophysiology of ODS due to the rapid correction of hypernatremia is poorly understood. In ODS caused by the rapid correction of hyponatremia, the reduced osmolality stimulates the movement of water from the extracellular matrix through the aquaporin channels embedded within the oligodendrocyte membranes into the cells. The cell expels electrolytes to compensate for the resulting swelling. A state of hemostasis is eventually attained, especially in cases of chronic hyponatremia (>48 hours), where the cell adjusts over time. However, with rapid correction of hyponatremia, the glial cells' capacity to replenish lost electrolytes is compromised, leading to cell demyelination [1].

The clinical presentation of ODS can be diverse, ranging from mild neurological symptoms to severe conditions such as ‘Locked-in’ syndrome [5]. Common symptoms include dysarthria, dysphagia, spastic quadriparesis, and altered mental status [5]. Diagnostic imaging, particularly MRI, plays a crucial role in identifying characteristic lesions and patterns of demyelination in the pontine and extrapontine regions of the brain [5]. Management of ODS focuses primarily on prevention and supportive care [1]. The exact prevalence of this condition is unknown due to the limited availability of epidemiological data in our region. 

Here, we report the case of a pregnant woman in her 30s who was discovered to be unresponsive at home and was brought to the hospital with features of severe sepsis, altered mental status, malnutrition, and dehydration. Her MRI brain revealed findings suggestive of Wernicke’s encephalopathy, with additional central pontine myelinosis. This case contributes to the evolving understanding of ODS in unique clinical scenarios, highlighting the complexity of the condition and the necessity for increased awareness of the disease, in addition to the utilization of appropriate diagnostic modalities and the adoption of a tailored approach to its treatment.

## Case presentation

A 35-year-old woman who was 16 weeks pregnant was brought to our ED by the ambulance. She was found unresponsive at home by the owner of the building. She appeared neglected, starved, dehydrated, and cachexic. Her clothes were soiled in urine and pus. As per the next of kin, she had a history of two vaginal deliveries and a cesarean section done six years ago. She was disoriented with unstable vitals and was taken to the resuscitation room as she was unresponsive.

A quick bedside scan showed a single live fetus with a biparietal diameter of 16 weeks, a fetal heart rate of 140, a normal amount of liquor around the fetus, and a closed cervix. On examination, there was a foul-smelling discharge from the vagina. Vaginal and nasal swabs were collected for viral screening.

Blood samples were collected for cultures and other lab tests. Her venous blood gas was suggestive of sepsis (high lactate) and acidosis (low bicarbonate). Her hemoglobin level was on the lower side, while her blood sugar levels were normal. She had a high white blood cell (WBC) count and high inflammatory markers. She also had abnormal electrolytes (prerenal kidney failure) and deranged liver function tests (acute liver injury).

The initial set of blood tests revealed abnormalities indicative of potential underlying pathologies. The patient presented with elevated serum sodium levels of 152 mmol/L (reference range: 136-145 mmol/L), along with elevated inflammatory markers, including C-reactive protein (CRP) at 44.9 mmol/L (reference range: 0.3-1.0 mmol/L), and an elevated WBC count of 14.3 × 10^9^/L (reference range: 4.5-11.0 × 10^9^/L). Procalcitonin levels were also elevated at 2.49 μg/L (reference range: <0.05 μg/L), consistent with the suspected diagnosis of Wernicke-Korsakoff encephalopathy. Additionally, the patient presented with a low hemoglobin level of 11.8 g/dL (reference range: 12.1-15.1 g/dL), as shown in Table 1.

**Table 1:** Laboratory parameters Na: sodium, K: potassium, Ca: calcium, WBC: white blood cells.

Laboratory parameters		
Serum data	Value	Normal range
Glycaemia (mg/dL)	153	70–110
Na^+^ (mmol/L)	152	135–146
K^+^ (mmol/L)	4.2	3.5–5.0
Ca^++ ^(mmol/L)	11.2	2.18–2.58
C-reactive protein (mmol/L)	44.9	0.3–1.0
Uric acid (mg/dL)	12.5	3.5–7.2
Creatinine (mg/dL)	3.1	0.6–1.1
Urea (mg/dL)	200	5–20
Total protein (g/dL)	8.3	6.0–8.3
Albumin (g/dL)	3.6	3.4–5.4
Anion gap (mEq/L)	25	4–12
Bicarbonate (HCO_3_) (mEq/L)	14.6	22–26
Bilirubin total (mg/dL)	2	<1.2
Procalcitonin (μg/L)	2.49	<0.05
WBC (× 10^9^/L)	14.3	4.5–11.0

**Figure 1:**
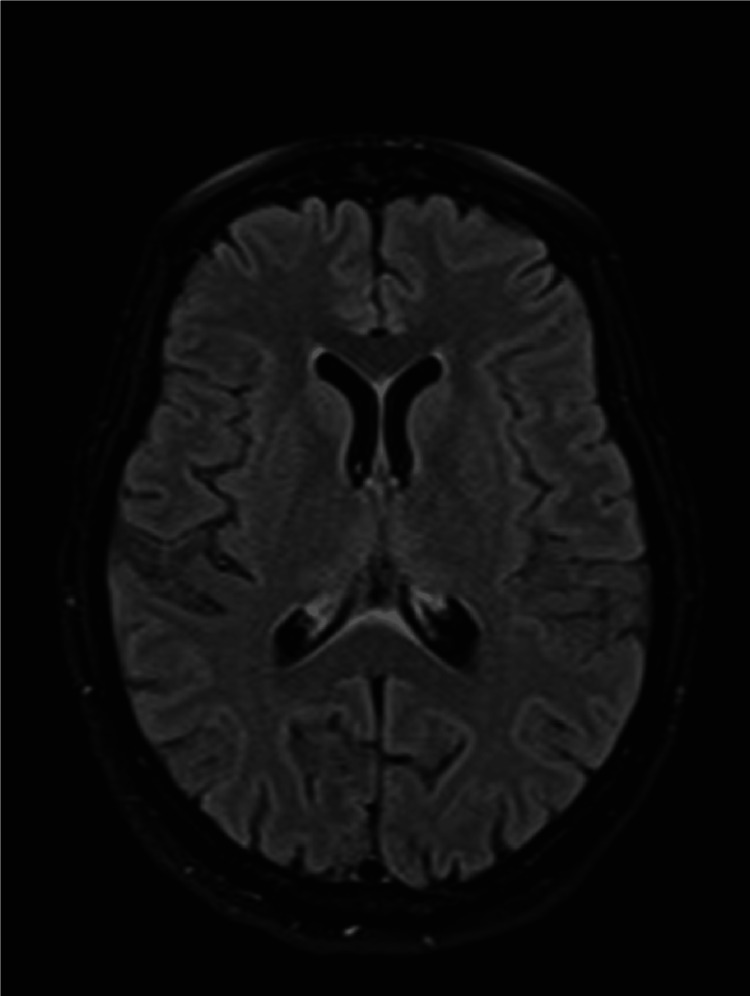
Brain MRI Bilateral symmetric abnormal increased signal intensity on T2/FLAIR sequences in various brain regions, including the mammillary bodies, dorsomedial thalami, tectal plate of the midbrain, periaqueductal grey matter, dorsal aspect of medulla oblongata, and minimally around the third ventricle. Similar signal changes are seen in bilateral frontal cortical grey matter and the central area of the pons with peripheral sparing; restricted diffusivity in specific brain regions; T1 hypointense abnormality; post-contrast enhancement in specific brain regions; basal ganglia and corpus callosum appeared normal; bilateral frontal nonspecific T2/FLAIR high signal spots within white matter; no intracranial collections or abnormal meningeal enhancement observed.

She was started on intravenous fluids and antibiotics. She was also started on nasogastric feeding as she complained of feeding difficulty and was subsequently shifted to the ICU. On re-examination the next day, the fetal heart sounds were found to be absent, and a diagnosis of septic miscarriage was made. The medical management of septic miscarriage was done by vaginal misoprostol, after which the fetus was expelled and the patient was taken for the evacuation of the uterus under anesthesia. Given her disorientation and altered mental status, a brain CT angiography with contrast was done under sedation, which turned out normal. She was then taken for an evacuation of the uterus under anesthesia after the expulsion of the products of conception. Following this, a brain MRI was done, which showed the following findings suggestive of Wernicke's encephalopathy and pontine osmotic demyelination syndrome (Figure 1).

She was then given intravenous vitamins B, C, and D as well as other supplements like calcium, magnesium and zinc and taken off of sedation, followed by extubation 48 hours later. The patient was once again started on nasogastric feeding as she continued to experience difficulty feeding; however, this time, she was able to tolerate small sips of water orally.

**Figure 2:**
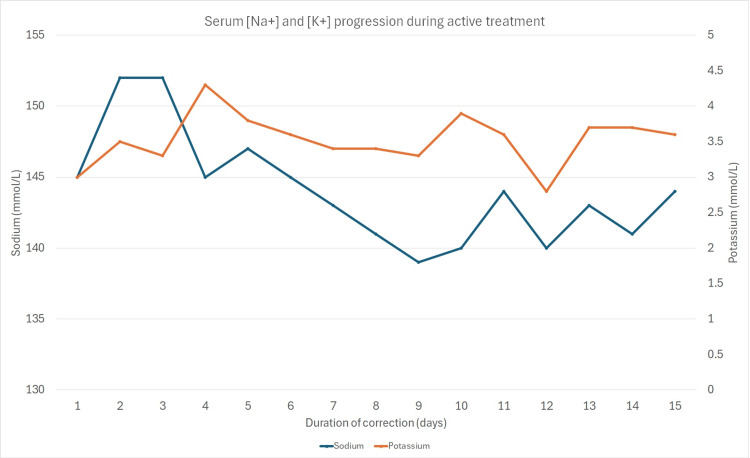
Serum [Na+] and [K+] progression during active treatment for the correction of severe hyponatraemia and hypokalemia in the patient Na: Sodium; K: Potassium.

The next day, she was transferred under the care of the medical team for further management as she continued experiencing weakness in her upper and lower limbs and feeding difficulty despite improvements in her renal and liver parameters and platelet count. She was continued on nasogastric feeding and started on physiotherapy. Daily serial electrolyte measurements were conducted to monitor the patient's response to treatment, aiming to normalize the electrolyte levels. These investigations were essential for evaluating the effectiveness of the administered medications and adjusting the treatment plan accordingly. Given her brain MRI results, she was seen by the neurology team and was given high doses of vitamins B and C, then shifted to oral vitamin B complex (Figure 2).

In the following days, she complained of right abdominal tenderness, for which an ultrasound was done that confirmed a stone in the common bile duct causing cholecystitis. The patient was given intravenous cefuroxime and metronidazole for seven days and was seen by the gastroenterologist, who recommended that there was no need for further endoscopic retrograde cholangiopancreatography (ERCP)/magnetic resonance cholangiopancreatography (MRCP) as her liver function test results were improving. A swallowing assessment was done, and she was started on a diet of soft foods. As her oral intake improved, she was taken off of the nasogastric feeding. The patient started complaining of right abdominal pain once again, and an ultrasound scan was repeated, which showed a dilated common bile duct with edematous pancreatitis. The surgical team advised her to continue on a soft diet and receive intravenous fluids. The gastroenterology team was re-consulted, and they performed an ERCP with stone removal and sphincterotomy. The patient remained stable post-procedure and could tolerate oral feeding without pain or vomiting.

## Discussion

The most scientific literature on osmotic demyelination syndrome revolves around the swift correction of hyponatremia while also considering the influence of other factors, such as pregnancy, chronic alcoholism, malnutrition, and liver transplantation, among various others, on its etiology. In the context of our case report, the etiology of ODS remains inconclusive; however, we hypothesize that it likely stemmed from prolonged malnutrition and dehydration, given the concomitant afflictions observed in the patient, including hypernatremia, anemia, Wernicke-Korsakoff syndrome, and hypoalbuminemia, all of which can be interconnected by malnutrition and dehydration.

In regard to the diagnosis of ODS, magnetic resonance imaging (MRI) is the most sensitive scan for detecting radiological changes. However, studies have shown that myelinolytic lesions in ODS may take several weeks to be detectable [6]. Given the positive MRI findings upon the admission of our patient, it is highly plausible that the onset of this syndrome occurred prior to her presenting to the hospital.

Imaging findings often show high signal intensity on T2-weighted images (T2WI) or low signal intensity on T1-weighted images (T1WI) at the affected site, which can be attributed to lesions demonstrating restricted diffusion [7]. A distinctive feature observed in ODS is the presence of a symmetrical, trident-shaped, high-intensity lesion within the pons on T2WI [8]. It was also found that in EPM, utilizing T2WI and FLAIR sequences, symmetrical high signal intensity lesions are noted bilaterally in the caudate nucleus and putamen [7], alongside involvement of other regions within the basal ganglia, thalami, and white matter. Additionally, in some cases of EPM, other anatomical sites may also be affected, including but not limited to the midbrain, gray matter, medulla oblongata, and cerebellum [9]. It is noteworthy that the corticospinal tracts, ventrolateral pons, and globus pallidus regions are typically spared in ODS [7].

To date, the only definitive approach to managing ODS involves providing supportive treatment aimed at alleviating the patient's symptoms while identifying and treating the underlying etiology. In all instances, it is crucial to gradually reduce the patient's sodium levels in order to prevent cerebral edema [5]. Although there are isolated cases documented in the literature where steroids, intravenous immunoglobulin, plasma exchange, and thyrotrophin-releasing hormone have been utilized in ODS treatment, no large-scale trials have been conducted to establish their efficacy. Therefore, further research is necessary before considering their incorporation into clinical practice [10].

Wernicke's encephalopathy is a neurological disorder characterized by an acute and reversible presentation that includes the classic triad of ataxic gait, ophthalmoplegia, and altered mental status [11]. This syndrome develops due to thiamine (vitamin B1) deficiency, which is primarily associated with alcoholism but can also result from inadequate intake, malnutrition, or an increased demand. Pregnancy represents a hypermetabolic state that increases the body's requirement for nutrients like thiamine, thereby making individuals more susceptible to deficiency [12]. While Wernicke's encephalopathy is a treatable condition, neglect and delayed treatment can lead to its progression into an irreversible chronic condition known as Korsakoff syndrome. Korsakoff syndrome is characterized by symptoms such as anterograde/retrograde amnesia, confabulation, hallucinations, personality changes, and disorientation. Collectively, these symptoms constitute Wernicke-Korsakoff syndrome [13].

Despite the significance of Wernicke's encephalopathy, it remains frequently underdiagnosed [14]. While the triad is characteristic, it is not pathognomonic, appearing in only 16% of cases [15]. Furthermore, patients often present with atypical symptoms, adding to the diagnostic ambiguity. This could increase the risk of underdiagnosis. Therefore, specific diagnostic tools such as MRI should be employed to allow for early detection and prompt management with vitamin B supplements.

MRI has consistently proven its efficacy as the golden standard for early detection and diagnosis of Wernicke encephalopathy. Despite a sensitivity of only 53%, the specificity of MR reaches 93%. T2 weighted imaging often reveals brain structures as hyperintense due to the edematous nature of the lesion. Key indicators of Wernicke’s include bilateral hyperintensities involving the mamillary bodies, periventricular gray matter, anterior and medial thalamic nuclei, and inferior and superior colliculi. Some studies even involved the cerebellum. FLAIR imaging was shown to be far superior compared to the traditional T2. In the case of a pregnant lady with hyperemesis gravidarum, high signal intensity was observed in the mamillary bodies and the hypothalamus. Additionally, several different cases revealed similar signals in periaqueductal gray matter, the third ventricle, the floor of the fourth ventricle, the anterior ventricular caps, and the medial thalami. Most of these findings are consistent with those observed in our patient, confirming the diagnosis of Wernicke's encephalopathy [16].

## Conclusions

Osmotic demyelination syndrome is a critical medical condition that requires immediate action. As seen in this case, pregnancy with malnutrition should be considered as one of the predisposing factors of osmotic demyelination syndrome. The occurrence of ODS in a pregnant patient, combined with sepsis and Wernicke's encephalopathy, is rare and poses a significant challenge to physicians in both diagnostic and therapeutic aspects. Another atypical presentation in such cases is the presence of hypernatremia rather than the typical hyponatremic correction of ODS. Fortunately, MRI aids in quicker diagnosis and allows for timely management before the condition progresses to a severe state. The management of such an uncommon and complex case is similar to the common presentations of ODS. Gradual correction of fluid and electrolyte imbalance is necessary for an optimum patient outcome.
